# Taipei Medical University Clinical Research Database: a collaborative hospital EHR database aligned with international common data standards

**DOI:** 10.1136/bmjhci-2023-100890

**Published:** 2024-05-14

**Authors:** Phung-Anh Nguyen, Min-Huei Hsu, Tzu-Hao Chang, Hsuan-Chia Yang, Chih-Wei Huang, Chia-Te Liao, Christine Y. Lu, Jason C. Hsu

**Affiliations:** 1 Clinical Data Center, Office of Data Science, Taipei Medical University, Taipei, Taiwan; 2 Research Center of Health Care Industry Data Science, College of Management, Taipei Medical University, Taipei, Taiwan; 3 Clinical Big Data Research Center, Taipei Medical University Hospital, Taipei Medical University, Taipei, Taiwan; 4 Office of Data Science, Taipei Medical University, Taipei, Taiwan; 5 Graduate Institute of Data Science, College of Management, Taipei Medical University, Taipei, Taiwan; 6 Graduate Institute of Biomedical Informatics, College of Medical Science and Technology, Taipei Medical University, Taipei, Taiwan; 7 International Center for Health Information Technology, College of Medical Science and Technology, Taipei Medical University, Taipei, Taiwan; 8 Research Center of Big Data and Meta-Analysis, Wan Fang Hospital, Taipei Medical University, Taipei, Taiwan; 9 Division of Nephrology, Department of Internal Medicine, Shuang Ho Hospital, Taipei Medical University, New Taipei City, Taiwan; 10 Division of Nephrology, Department of Internal Medicine, School of Medicine, College of Medicine, Taipei Medical University, Taipei, Taiwan; 11 Taipei Medical University-Research Center of Urology and Kidney, Taipei Medical University, Taipei, Taiwan; 12 Department of Population Medicine, Harvard Medical School and Harvard Pilgrim Health Care Institute, Boston, MA, USA; 13 Kolling Institute, Faculty of Medicine and Health, The University of Sydney and the Northern Sydney Local Health District, Sydney, NSW, Australia; 14 School of Pharmacy, Faculty of Medicine and Health, The University of Sydney, Sydney, NSW, Australia; 15 International Ph.D. Program in Biotech and Healthcare Management, College of Management, Taipei Medical Unversity, Taipei, Taiwan

**Keywords:** database management systems, evidence-based medicine, health information management, Information Management, Medical Record Linkage

## Abstract

**Objective:**

The objective of this paper is to provide a comprehensive overview of the development and features of the Taipei Medical University Clinical Research Database (TMUCRD), a repository of real-world data (RWD) derived from electronic health records (EHRs) and other sources.

**Methods:**

TMUCRD was developed by integrating EHRs from three affiliated hospitals, including Taipei Medical University Hospital, Wan-Fang Hospital and Shuang-Ho Hospital. The data cover over 15 years and include diverse patient care information. The database was converted to the Observational Medical Outcomes Partnership Common Data Model (OMOP CDM) for standardisation.

**Results:**

TMUCRD comprises 89 tables (eg, 29 tables for each hospital and 2 linked tables), including demographics, diagnoses, medications, procedures and measurements, among others. It encompasses data from more than 4.15 million patients with various medical records, spanning from the year 2004 to 2021. The dataset offers insights into disease prevalence, medication usage, laboratory tests and patient characteristics.

**Discussion:**

TMUCRD stands out due to its unique advantages, including diverse data types, comprehensive patient information, linked mortality and cancer registry data, regular updates and a swift application process. Its compatibility with the OMOP CDM enhances its usability and interoperability.

**Conclusion:**

TMUCRD serves as a valuable resource for researchers and scholars interested in leveraging RWD for clinical research. Its availability and integration of diverse healthcare data contribute to a collaborative and data-driven approach to advancing medical knowledge and practice.

**WHAT IS ALREADY KNOWN ON THIS TOPIC**

- Existing knowledge encompasses the increasing use of digital solutions in healthcare, the importance of real-world data (RWD) for generating real-world evidence, and the limitations of traditional clinical trials with limited participant diversity.

**WHAT THIS STUDY ADDS**

- This study presents the development and features of the Taipei Medical University Clinical Research Database (TMUCRD), highlighting its extensive collection of RWD spanning multiple hospitals over a decade. TMUCRD provides valuable insights into patient medical records, underscoring its role as a robust platform for collaborative research and evidence-driven healthcare improvements.

**HOW THIS STUDY MIGHT AFFECT RESEARCH, PRACTICE OR POLICY**

- This study’s establishment of the TMUCRD will significantly impact research by providing a rich source of RWD for diverse healthcare investigations. It has the potential to enhance evidence-based medical practices and inform healthcare policies by facilitating collaborative research efforts and promoting data-driven decision-making in the medical field.

## Introduction

The adoption of various digital solutions in healthcare, especially in US hospitals, has significantly increased, going from 6.6% to 81.2% for electronic health records (EHRs) and from 3.6% to 63.2% for comprehensive systems in recent times.^1–3^ It has become increasingly important to gather solid evidence and understanding to incorporate these digital solutions into regular medical practices.^4 5^ This shift towards digitalisation has the potential to provide patients and medical professionals with effective tools to achieve health-related goals.^6 7^ Notably, this trend has gained recognition from regulatory bodies like the US Food and Drug Administration^8^ and international health organisations such as the WHO.^9^

Digital systems for managing health information play a crucial role in systematically collecting high-quality and trustworthy data.^10^ Being able to make informed decisions based on data, especially real-world data (RWD), is vital for healthcare providers striving to deliver top-notch care.^11^ RWD comes from various sources including electronic medical records (EMRs), databases for insurance claims and billing, disease registries and wearable devices. By using strong analytical methods on RWD, tangible real-world evidence (RWE) can be generated, which holds significant potential for improving health outcomes and patient well-being.^12–14^ RWE provides a notable contrast between expected outcomes and actual observations, especially when compared with traditional clinical trials that often have limitations due to their narrow participant groups, making it challenging to apply findings to broader populations.^15–18^ RWE studies are becoming an effective approach for postmarket surveillance, offering valuable additional evidence, particularly for identifying rare adverse events and long-term effects of established medications. However, relying on a single RWD source might not yield sufficiently strong evidence for healthcare providers, decision-makers or key opinion leaders, particularly in healthcare. This effectiveness would come from their ability to analyse large groups of patients over extended periods from diverse RWD sources.^19 20^ Challenges arise due to differing data formats across clinical settings and potential disparities in data processing steps, even when following study protocols. To address these challenges comprehensively, adopting a common data model (CDM) emerges as a potential solution, supporting global research strategies.

Taipei Medical University (TMU) has successfully integrated EMRs from its three affiliated hospitals—Taipei Medical University Hospital (TMUH), Wan-Fang Hospital (WFH) and Shuang-Ho Hospital (SHH))—with external data sources supplied by the Taiwan government. This integration has led to the creation of the Taipei Medical University Clinical Research Database (TMUCRD) in 2015. This paper aims to thoroughly describe the development of TMUCRD, a comprehensive repository of RWD. The dataset covers more than 15 years and contains detailed information about individual patient care experiences. This database serves as a valuable tool for advancing clinical research, sharing knowledge and collaborating with scholars, organisations and industries worldwide.

## Methods

**Figure 1:**
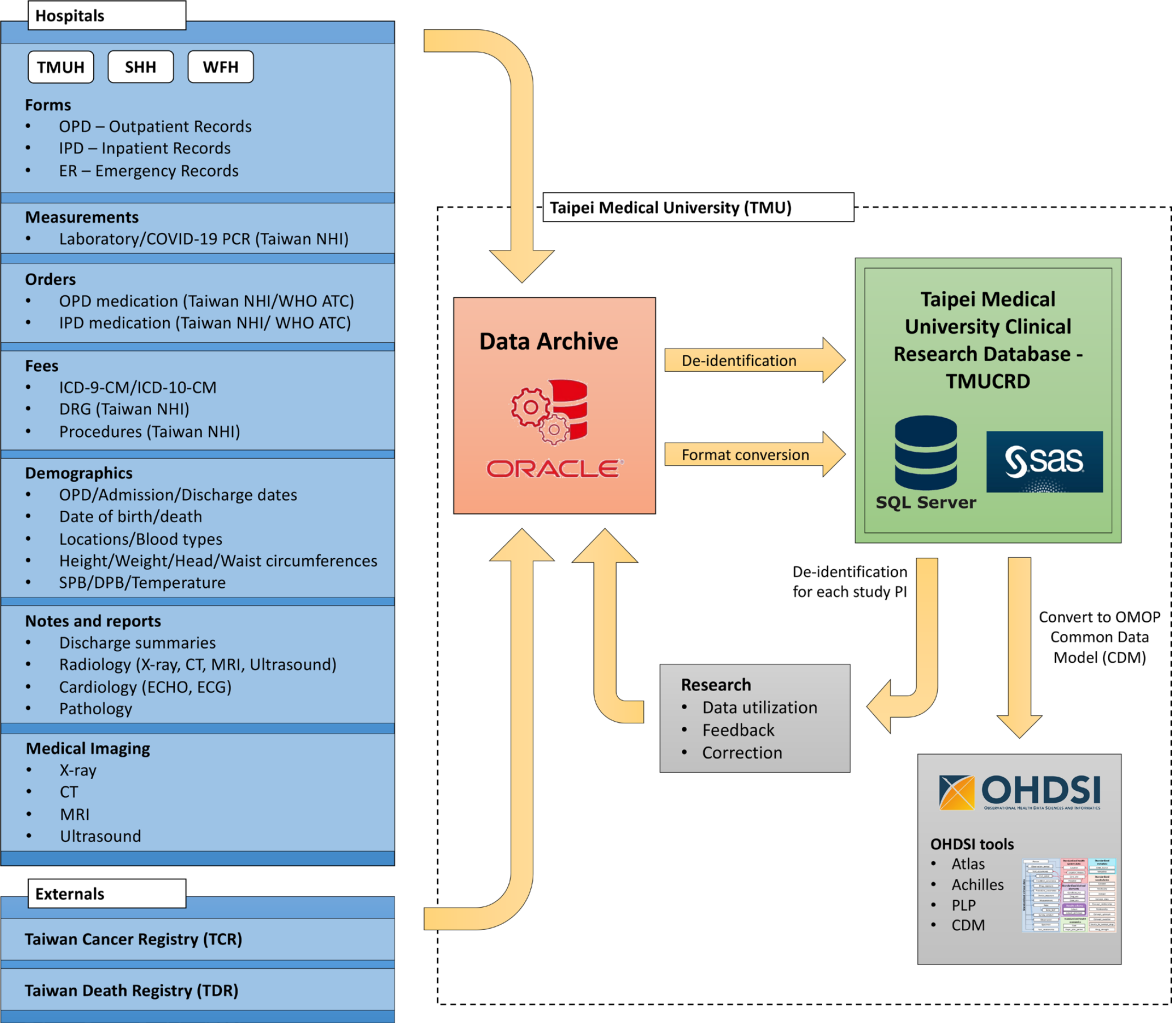
The overview of the TMUCRD. ATC, anatomical therapeutic chemical; ICD-9-CM, International Classification of Disease, 9th Revision, Clinical Modification; NHI, National Health Insurance; OHDSI, Observational Health Data Sciences and Informatics; SHH, Shuang Ho Hospital; TMUH, Taipei Medical University Hospital; WFH, Wan-Fang Hospital.

The TMUCRD is a central data warehouse of EHRs, providing us with a platform to leverage our accumulated expertise in managing and combining data, as illustrated in figure 1. This data repository contains a wealth of information including details about patients’ demographics, observations, diagnoses, prescribed medications, medical devices used, laboratory measurements, procedure codes, pathology and medical imaging reports, as well as vital health data. Currently, the database covers a vast range of medical records for approximately 4.15 million patients, spanning from the year 2004 to 2021.

### Database development

The Clinical Data Centre (CDC) at the TMU Office of Data Science is a collaborative group made up of experts in data science, pharmacists and practising physicians. They have joined forces to create the research database. The TMUCRD database is filled with information collected during regular hospital care, meaning it does not cause any extra work for healthcare providers or disrupt their usual routines. The data have been gathered from various sources and linked, including:

- Archives from hospital information system (HIS) databases.
- Taiwan Cancer Registry database.
- Taiwan Death Registry database.

During the data collection period, information was gathered from three distinct HIS—TMUH, WFH and SSH. These systems served as the origin of clinical data, comprising various elements such as:

- Different types of forms like outpatient, inpatient and emergency records.
- Results of ordered measurements.
- Medications prescribed by clinicians/physicians.
- Details about procedures performed and associated fees.
- Patient demographic data including birthdates, zip codes, height, weight, blood pressure readings (systolic blood pressure, diastolic blood pressure), temperature for each hospital visit and in-hospital mortality.
- Recorded notes such as discharge summaries and reports from examinations such as radiology, cardiology and pathology.
- Medical images in Digital Imaging and Communications in Medicine (DICOM) format, which include X-rays, CT scans, MRIs and ultrasounds.

With the exception of data specifically collected for research purposes, the data were extracted and organised into database tables with structures distinct from those of the HISs. These data are stored individually for each hospital and are differentiated using a suffix denoting their source. For instance, TMUH’s outpatient visits are stored in the OPD_BASIC_T table while WFH’s and SHH’s outpatient visits are stored in the OPD_BASIC_W and OPD_BASIC_S tables, respectively. However, patient data can still be cross-referenced across hospitals using their pseudoidentification, represented by the ‘ID_NO’.

We acquired information about mortality occurring outside the hospital environment by referring to the Taiwan Death Registry database, which is maintained by the Taiwan Ministry of the Interior.^21^ Additionally, we have established a link between the TMUCRD and the Taiwan Cancer Registry, a dataset offered by the Taiwan Ministry of Health.^22^ This linkage allowed us to identify patients who were diagnosed with various forms of cancer and had visited any of the three hospitals in our study.

The TMUCRD vocabulary contains various terms, and the team at the CDC has worked to link these terms with standardised dictionaries within the database. As an example, the codes used for laboratory tests and medications in TMUCRD, which are recognised by Taiwan’s National Health Insurance (NHI), have been connected to codes in LOINC^23^ and RxNorm,^24^ respectively. These efforts have been made to adapt TMUCRD into widely accepted data formats, such as the Observational Medical Outcomes Partnership CDM (OMOP CDM). This adaptation enables the use of consistent tools and methodologies.^25^

### Deidentification

Prior to being integrated into the TMUCRD database, the data underwent a deidentification process to adhere to the standards set by the Health Insurance Portability and Accountability Act (HIPAA). The initial step was conducted independently by the Centre for Management and Development (CMD) at TMU.^26^ This deidentification was achieved using structured data techniques.^27^ The process for structured data involved the elimination of eighteen specific data elements that could potentially identify individuals, as outlined in HIPAA. This removal included details such as patient names, phone numbers, addresses and dates. Notably, for the birth dates, only the year and month were retained for each patient, ensuring further privacy.

Moreover, an additional layer of deidentification was implemented by introducing randomisation to the variables within each data table. Essentially, we combined the initial pseudoidentification with a randomly generated salt-key, which consists of data from one or multiple variables associated with each patient. This salt-key serves as an additional input to a one-way function that hashed the pseudoidentification. Additionally, we employed checksum functions using MD5, SHA1 and SHA256 algorithms, which are types of hash functions. This process was completed before providing the data to each respective study principal investigator (PI). It is important to note that the components of this deidentification system are consistently expanded to accommodate new data as it is obtained.

The code used to create the TMUCRD introduction website and its accompanying documentation is accessible solely to individuals associated with TMU, including the PIs. The link to access this code is available.^28^

### CDM conversion

The OMOP CDM serves as a standardised structure for organising observational medical data. Its purpose is to ensure the reliable analysis and utilisation of medical information for research purposes. This model includes standardised vocabularies that establish uniform terminology usage across different medical areas.^29^ Essentially, it provides a systematic framework for converting varied healthcare data into a shared format, facilitating consistent analysis across diverse data sources and research investigations.^30^ Starting in January 2021, the TMUCRD database embarked on a journey to adapt its data to the OMOP CDM standard. This transition was facilitated with the support of the Observational Health Data Sciences and Informatics (OHDSI) global initiative. The amalgamation of data from all three affiliated hospitals led to the naming of the database as the TMU-CMD.

### Technical validation

To maintain the close representation of the original data collected from the three affiliated hospitals, we aimed to minimise significant changes to the structure of TMUCRD while achieving the necessary level of deidentification and data schema.

We adhered to the best practices in scientific computing whenever feasible. The development of TMUCRD was managed with version control, ensuring that changes were well tracked and documented. Issue tracking was implemented to transparently document any limitations in the data or code and address them appropriately. We actively encourage the research community to report and address any issues they come across. Furthermore, we have established a system for minor updates to the database.

The process of converting to TMU-CDM, which is the TMU-CDM, was carefully validated. This validation process followed the guidance of the OHDSI global initiative, particularly the SOS project.^31^ This rigorous approach ensured the accuracy and reliability of the conversion process.

## Results

### Data records and tables

TMUCRD is a relational database that comprises 29 individual tables for each of the three hospitals involved. Additionally, there are two linked tables that connect with the Taiwan government (online supplemental appendix figure S1). Within each hospital, these tables are connected using identifiers, typically employing hospital-specific and visit-specific IDs (eg, CHR_NO, FEE_NO) for each patient. For example, ‘CHR_NO’ refers to a unique patient in a hospital, and ‘CHR_NO and FEE_NO’ refer to a unique outpatient visit or a unique admission to the hospital.

10.1136/bmjhci-2023-100890.supp1 Supplementary data

To ensure the accuracy of transformations and to maintain the fidelity of the original hospital data, we were cautious not to make assumptions about the underlying data. This approach enabled TMUCRD to faithfully represent the raw hospital data. The distribution of data across different categories and tables is outlined in table 1. Broadly, TMUCRD encompassed nine categories: demographics, diagnoses, medications, procedures, measurements, image examinations and radiology, surgeries, cancer-related data, pathology and vocabulary. The vocabulary category contained dictionary tables providing definitions for various identifiers. For instance, in the OPD_MED table, each row was associated with a unique MED_CODE representing a medication concept as outlined by Taiwan’s NHI regulations. By connecting the OPD_MED and MED_BASIC tables using MED_CODE, we could discern the concept behind a specific MED_CODE.

**Table 1:** Overview of the TMUCRD table data

Category/table name	Description
Demographics	
CHR_BASIC	Basic demographic characteristic of unique patients in the database (defines ID_NO)
NDR_CASE	Patients and their cause of death registered by the Taiwan Ministry of Health and Welfare linked to the database
BIO_INF	Vital signs recorded for a given patient in different visits
Diagnosis	
OPD_BASIC	Patients’ diagnosis at outpatient departments (defines FEE_NO)
IPD_BASIC	Patients’ diagnosis at admissions (defines FEE_NO)
CHR_ICD	Summary information of patients discharge from admissions
EPD_HIS	Patients’ diagnosis at emergency department (defines FEE_NO)
Medication	
OPD_MED	Medication order for a given patient at the outpatient department (defines FEE_NO)
OPD_WARNING	Drug allergy recorded of patients at the outpatient department
UD_ORDER	Medication order for a given patient at admissions (defines FEE_NO)
UD_ORDER_LOG	Drug uses recorded of patients at admissions
Procedure	
OPD_FEE	Procedure orders for a given patient at the outpatient department, using local hospital codes
IPD_FEE	Procedure orders for a given patient at admissions, using local hospitals codes
Measurement	
OPD_EXPER/EXPER_ORDER	Laboratory measurement orders for patients both at the outpatient and admissions
LABRESULT/EXPER_SIGN	Laboratory measurement values for patients both at the outpatient and admissions
Image examination and radiology	
LAB_SCHE	Examination orders for patients both at the outpatient and admissions
CARDIAC_ECHO_REPORT	All recorded echocardiography report information for a given patient
X_RAY	All recorded X-Ray report information for a given patient
RAD_REPORT	All recorded radiographic imaging report information for a given patient
MMSE	All recorded mental examination report information for a given patient
Surgery	
OP_BASIC	All recorded surgery report information for a given patient (except nursing reports)
Cancer	
CR_TCASE	Patients and their cancer information registered by the Taiwan Ministry of Health and Welfare linked to the database
Pathology	
PATT_REPORT	All recorded pathology report information for a given patient
Vocabulary	
FEE_BASIC	Dictionary of the procedure, laboratory and other Item codes, including local hospitals and Taiwan National Health Insurance (NHI) codes
ICD9_BASIC	Dictionary of the International Statistical Classification of Diseases and Related Health Problems, 9th revision codes relating to diagnosis
ICD10_BASIC	Dictionary of the International Statistical Classification of Diseases and Related Health Problems, 10th revision codes relating to diagnosis
MED_BASIC	Dictionary of the Medication codes, including local hospitals and Taiwan NHI codes
EXPERIMENT/EXPER_REFERDATA	Dictionary of the examination codes, including local hospitals and Taiwan NHI codes
BED_BASIC	Information on the different types of beds in the database
DEPT_BASIC	Information on the different departments in the database
DOC_BASIC	Information on every physician and pharmacist who had recorded data in the database

TMUCRD, Taipei Medical University Clinical Research Database.

Furthermore, information pertaining to patient visits was stored in the diagnosis category through tables such as OPD_BASIC, IPD_BASIC and EPD_BASIC. Other categories contained data linked to patient treatments, medications, procedures, measurements, and image examinations and radiology. In some cases, it was possible to merge tables. For instance, the OPD_FEE and OPD_EXPER tables both contained details about measurements and could be combined. However, we chose to maintain the independent tables for clarity, given the substantial disparities in data sources and content.

### Patient demographic characteristics

TMUCRD encompassed data from a 4.15 million unique patients who visited three hospitals in northern Taiwan from 2004 to 2021. This data compilation involved more than 61.5 million outpatient visits, around 3 million emergency visits, and roughly 1.1 million hospital admissions. The average age of patients during their initial visit had a mean of 38.8 years with an SD of 20, and a median of 37.2 years within an IQR range of 24.5–53.2 years. A majority of the patients were female, making up 53.9% of the total. The overall TMUCRD dataset showed a mortality rate of 7.5%, as linked with the National Death Registry, while the in-hospital mortality rate was 0.88%. On average, patients spent around 4 days in the hospital (eg, with a median of 4 days and an IQR range of 2–8 days). The mean observation period for patients was approximately 1451 days, with an SD of 1940 days. For a detailed breakdown of the patient population within each hospital, it can refer to table 2.

**Table 2:** Details of the TMUCRD patient demographic characteristics by first visit

	WFH	SHH	TMUH	Overall
Data period	January 2004–December 2021	June 2008–December 2021	December 2011–December 2021	–
Distinct patients, N (%)	1 708 838 (41.1)	1 507 456 (36.3)	1 812 468 (43.6)	4 155 674 (100)
Outpatient clinics, N (%)	24 646 028 (40.1)	20 704 319 (33.7)	16 153 474 (26.3)	61 503 821 (100)
Emergency, N (%)	1 101 787 (36.9)	1 329 296 (44.5)	553 901 (18.6)	2 984 984 (100)
Hospital admissions, N (%)	354 941 (31.5)	469 096 (41.6)	303 415 (26.9)	1 127 452 (100)
Hospital length of stay, days				
Mean (SD)	9 (61.3)	7 (26.1)	7 (45.1)	8 (44.9)
Median (IQR)	4 (2–8)	4 (3–8)	3 (2–6)	4 (2–8)
Age at the first visit, years				
Mean (SD)	37.3 (19.8)	40.6 (20.1)	40.4 (18.8)	38.8 (20.0)
Median (IQR)	35.0 (22.9–51.1)	40.2 (26.5–55.6)	38.6 (27.7–53.8)	37.2 (24.5–53.2)
Gender, N (%)				
Female	891 090 (39.8)	820 566 (36.7)	1 027 409 (45.9)	2 237 884 (53.9)
Male	817 753 (42.7)	682 644 (35.6)	784 162 (40.9)	1 915 264 (46.1)
Mortality, N (%)				
Overall (NDR)	135 507 (43.6)	79 121 (25.4)	145 821 (46.9)	311 004 (7.5)
Hospital mortality	15 238 (41.8)	13 742 (37.7)	7510 (20.5)	36 488 (0.88)
Observation period, days				
Mean (SD)	1444 (2100)	1161 (1695)	780 (1237)	1451 (1940)
Median (IQR)	155 (1–2363)	196 (1–2107)	85 (1–1265)	419 (1–2607)

NDR, National Death Registry; SHH, Shuang Ho Hospital; TMUCRD, Taipei Medical University Clinical Research Database; TMUH, Taipei Medical University Hospital; WFH, Wan-Fang Hospital.

### Diseases

Table 3 presents information concerning disease categories within each hospital. A total of 19 disease systems, categorised using the International Classification of Disease, 9th and 10th Revision, Clinical Modification (ICD-9-CM and ICD-10-CM), were subjected to descriptive analysis. Without considering the analysis of symptoms, signs, ill-defined conditions (ie, ICD9: 780–799; ICD10: P00–R99), and supplementary classifications encompassing factors influencing health status and healthcare interactions (ie, ICD9: V01–V91; ICD10: U00–U85, Z00–Z99); the three most prevalent disease categories, were as follows:

- Diseases of the digestive system (ie, ICD9: 520–579; ICD10: K00–K95), which constituted 26.7% of all patients.
- Diseases of the musculoskeletal system and connective tissue (ie, ICD9: 710–739; ICD10: M00–M99), accounting for 21.2% of all patients.
- Diseases of the respiratory system (ie, ICD9: 460–519; ICD10: J00–J99), representing 20.4% of all patients.

**Table 3:** Distribution of diseases systems by ICD and the medication classes by ATC codes

	WFH, n (%) n=1 708 838 (41.1)	SHH, n (%) n=1 507 456 (36.3)	TMUH, n (%) n=1 812 468 (43.6)	Overall, n (%) n=4 155 674 (100)
(A) Disease systems (ICD-9-CM; ICD-10-CM codes)				
Infectious and parasitic diseases (ICD9: 001–139; ICD10: A00–B99)	188 239 (11)	150 855 (10)	96 605 (5.3)	427 637 (10.3)
Neoplasms (ICD9: 140–239; ICD10: C00–D49)	192 219 (11.2)	165 407 (11)	190 239 (10.5)	524 757 (12.6)
Endocrine, nutritional and metabolic siseases, and immunity disorders (ICD9: 240–279; ICD10: D50–D78)	175 917 (10.3)	175 667 (11.7)	192 568 (10.6)	526 785 (12.7)
Diseases of the blood and blood-forming organs (ICD9: 280–289; ICD10: D80–D89, E00–E89)	132 502 (7.8)	170 113 (11.3)	165 837 (9.1)	457 048 (11)
Mental disorders (ICD9: 290–319; ICD10: F01–F99)	126 526 (7.4)	115 707 (7.7)	82 543 (4.6)	315 086 (7.6)
Diseases of the nervous system and sense organs (ICD9: 320–389; ICD10: G00–G99, H00–H59, H60–H95)	291 972 (17.1)	294 077 (19.5)	188 926 (10.4)	745 191 (17.9)
Diseases of the circulatory system (ICD9: 390–459; ICD10: I00–I99)	223 187 (13.1)	228 224 (15.1)	168 545 (9.3)	593 200 (14.3)
Diseases of the respiratory system (ICD9: 460–519; ICD10: J00–J99)	332 493 (19.5)	351 593 (23.3)	194 414 (10.7)	847 520 (20.4)
Diseases of the digestive system (ICD9: 520–579; ICD10: K00–K95)	383 595 (22.4)	464 523 (30.8)	312 873 (17.3)	1 107 945 (26.7)
Diseases of the genitourinary system (ICD9: 580–629; ICD10: N00–N99)	256 598 (15)	284 844 (18.9)	243 678 (13.4)	750 833 (18.1)
Complications of pregnancy, childbirth and the puerperium (ICD9: 630–679; ICD10: O00–O9A)	25 429 (1.5)	21 266 (1.4)	19 985 (1.1)	65 014 (1.6)
Diseases of the skin and subcutaneous tissue (ICD9: 680–709; ICD10: L00–L99)	266 921 (15.6)	230 615 (15.3)	147 242 (8.1)	625 496 (15.1)
Diseases of the musculoskeletal system and connective tissue (ICD9: 710–739; ICD10: M00–M99)	348 092 (20.4)	340 553 (22.6)	245 354 (13.5)	882 368 (21.2)
Congenital anomalies (ICD9: 740–759; ICD10: Q00–Q99)	27 730 (1.6)	19 710 (1.3)	15 338 (8.5)	61 809 (1.5)
Certain conditions originating in the perinatal period (ICD9: 760–779; ICD10: P00–P96)	12 661 (0.7)	10 551 (0.7)	6603 (0.4)	29 726 (0.7)
Symptoms, signs and Ill-defined conditions (ICD9: 780–799; ICD10: R00–R99)	393 477 (23)	508 424 (33.7)	302 514 (16.7)	1 151 059 (27.7)
Injury and poisoning (ICD9: 800–999; ICD10: S00–T88)	323 758 (18.9)	344 203 (22.8)	172 029 (9.5)	809 761 (19.5)
Supplementary classification of factors influencing health status and contact with health services (ICD9: V01–V91; ICD10: U00–U85, Z00–Z99)	580 196 (34)	804 887 (53.4)	531 065 (29.3)	1 752 129 (42.1)
Supplementary classification of external causes of injury and poisoning (ICD9: E000–E999; ICD10: V00–Y99)	9543 (0.6)	34 116 (2.3)	14 484 (0.8)	57 896 (1.4)
(B) Medications classes, ATC first level				
A—Alimentary tract and metabolism	450 974 (26.4)	562 247 (37.3)	348 081 (19.2)	1 289 116 (31)
B—Blood and blood-forming organs	366 035 (21.4)	419 266 (27.8)	245 904 (13.6)	986 264 (23.7)
C—Cardiovascular system	285 721 (16.7)	312 045 (20.7)	210 058 (11.6)	774 258 (18.6)
D—Dermatological	329 324 (19.3)	315 405 (20.9)	199 766 (11)	814 951 (19.6)
G —Genito urinary system and sex hormones	139 966 (8.2)	135 487 (9)	114 848 (6.3)	378 449 (9.1)
H—Systemic hormonal preparations, excluding sex hormones and insulins	194 446 (11.4)	242 853 (16.1)	162 001 (8.9)	581 247 (14)
J—Anti-infectives for systemic use	425 757 (24.9)	470 468 (31.2)	293 058 (16.2)	1 136 587 (27.4)
L—Antineoplastic and immunomodulating agents	17 869 (1.1)	22 568 (1.5)	30 055 (1.7)	69 149 (1.7)
M—Musculoskeletal system	552 981 (32.4)	636 300 (42.2)	365 197 (20.1)	1 459 426 (35.1)
N—Nervous system	579 776 (33.9)	627 858 (41.7)	391 018 (21.6)	1 497 538 (36)
P—Antiparasitic products, insecticides and repellents	30 615 (1.8)	31 425 (2.1)	20 125 (1.1)	81 281 (2)
R—Respiratory system	410 430 (24)	471 027 (31.2)	254 361 (14)	1 086 966 (26.2)
S—Sensory organs	246 766 (14.4)	192 156 (12.7)	122 779 (6.8)	547 778 (13.2)
V—Various	71 841 (4.2)	99 369 (6.6)	10 173 (0.6)	180 267 (4.3)

ATC, anatomical therapeutic chemical; ICD, International Classification of Disease; ICD-9-CM, ICD, 9th Revision, Clinical Modification; SHH, Shuang Ho Hospital; TMUH, Taipei Medical University Hospital; WFH, Wan-Fang Hospital.

### Medications

Medications were organised into different groups based on the specific organ or system they impact, categorised at various levels. In table 3B, we can observe the prevalence rates of 14 distinct medication groups, classified using the Anatomical Therapeutic Chemical classification system at the first level. The majority of medications utilised fell within class N (nervous system), constituting 36% of usage. Following closely were class M (musculoskeletal system), class A (alimentary tract and metabolism) and class J (anti-infective for systemic use), accounting for 35.1%, 31% and 27.4% of usage, respectively.

### Laboratory types

**Figure 2:**
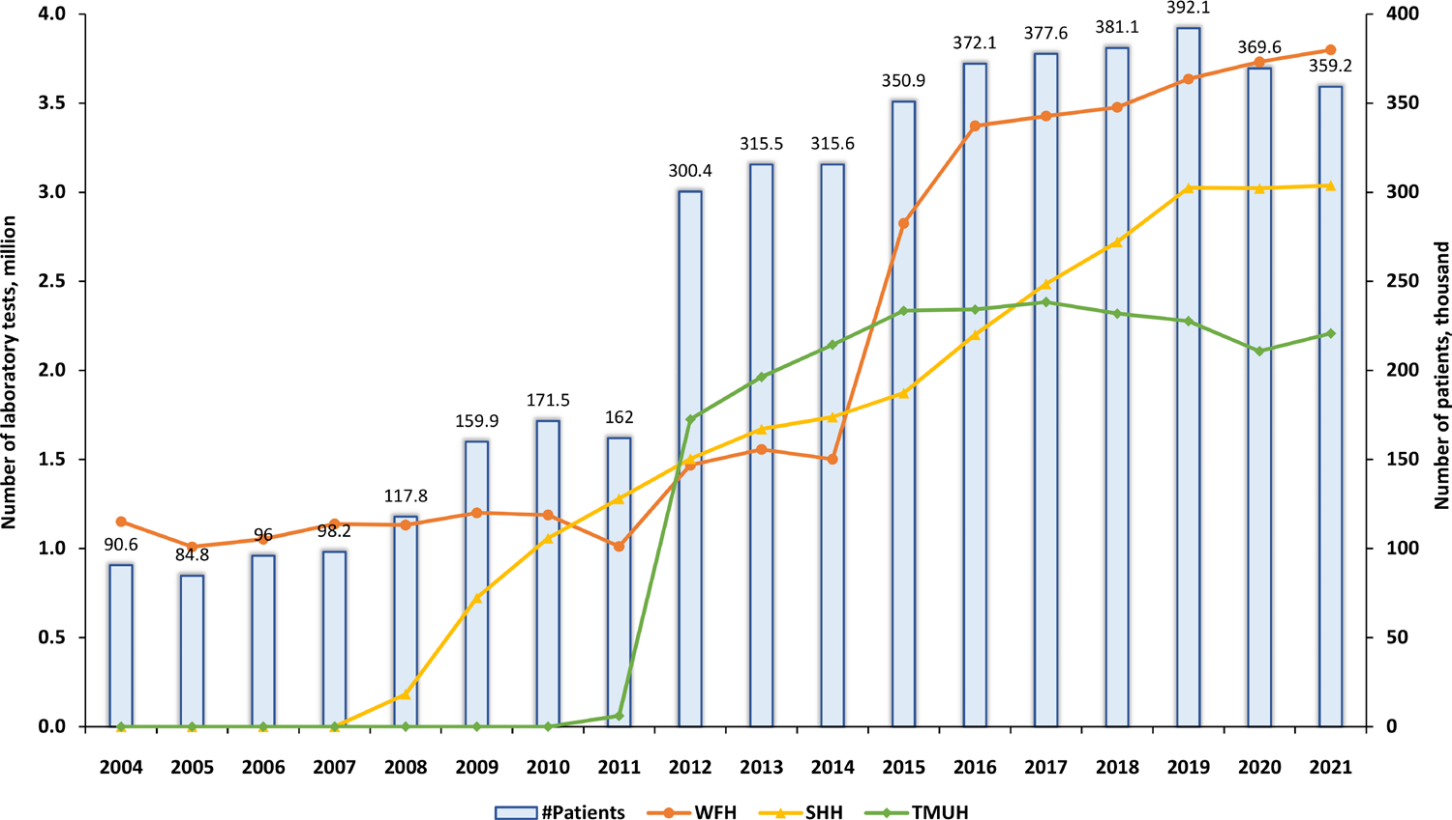
The overview of the number of laboratory tests over. SHH, Shuang Ho Hospital; TMUH, Taipei Medical University Hospital; WFH, Wan-Fang Hospital.

Figure 2 presents data regarding the count of laboratory tests conducted in the outpatient department, categorised by years, for each individual hospital within the TMUCRD. Additionally, the total count of patients who underwent these tests is also displayed. The range of laboratory tests varied widely, spanning from 1.15 to 3.8 million over the course of 18 years at WFH. Notably, the number of tests notably rose in 2021, reaching 3.04 million and 2.21 million for SHH and TMUH, respectively. For a more comprehensive breakdown of this information, it can refer to online supplemental appendix, table S1.

## Discussion

The Taiwan NHI database has gained significant recognition among medical researchers and scholars.^32–34^ However, TMUCRD offers several notable advantages, including:

- Multiple laboratory test data: It includes a wide array of laboratory test results. For instance, creatinine levels can serve as a basis for evaluating the severity of chronic kidney disease (CKD).
- Comprehensive pathology reports: Pathology reports are comprehensive, encompassing gene tests and other biomarker information.
- Detailed patient admission data: Information about patient admissions, covering surgeries, drug usage, timing and specifics of various treatments during hospitalisation, is available.
- Itemised records and services: It logs items and services that require patient payment, such as health checks and new drugs not yet covered by insurance.
- Linked with death registration files: TMUCRD has linked with the Ministry of Health and Welfare’s death registration records. This facilitates accurate information about patient deaths, including date and cause. This is particularly useful for cancer treatment and prognosis survival analysis.
- Linked with cancer registration data: Integration with the National Health Service’s cancer registration records provides additional insights into cancer treatment, significantly aiding cancer-related research.
- Regular data updates: Data are consistently updated for the ongoing year.
- Speedy application process: The application process is swift, including institutional review board (IRB) review time, with data obtainable within approximately 1–2 months.
- Fee exemption for TMU affiliates: Colleagues from TMU and its affiliated institutions are exempt from application fees.
- Data usage fee exemption for TMU scholars: TMU and its affiliated scholars enjoy exemption from data usage fees within their work areas.

These advantages collectively position TMUCRD as a valuable resource for researchers.

### Data access

TMUCRD is available in a range of specially structured SAS files. These files typically reside within SAS libraries and can be processed using SAS software V.9.4 (SAS Institute). Additionally, the data from these files can be imported into database systems like MySQL, PostgreSQL or MSSQL Server. Given that the database holds intricate information about patients’ clinical care, it necessitates appropriate handling with due care and respect. Researchers aiming to access the data must follow a formal procedure, outlined on the TMU-CDC website.^35^ There are specific prerequisites and steps to be fulfilled before access is granted:

- TMUCRD is accessible to PIs and research scholars affiliated with TMU and its associated hospitals.
- Applicants need to acquire and complete the research database application and case report forms.^36^ Subsequently, they must seek approval from the ethics committee through the TMU-eJIRB system.^37^ PIs, investigators and analytical personnel must be mentioned in the IRB and are required to sign the data use agreement. This approval process typically takes a minimum of 2 weeks. Once the application is sanctioned, the TMU-CDC will inform PIs and scholars via email, providing instructions for accessing the dataset.
- We provided two services:
  - ‘Data to go’: Applicants can receive released data to conduct their analyses. However, it noted that the number of patients included is less than 1% of the total population (approximately 30 000 patients), and no reports, such as radiology, pathology and discharge summaries, are provided.
  - ‘Report to go’: Applicants can access various types of data and can analyse their study research in a designated ‘clean room’. In this setting, individuals are prohibited from bringing in any external devices or items, ensuring that only verified reports intended for publication can be carried out.

### Example usage

Since the TMUCRD became available for application in January 2020, the Data Office has received and successfully managed 289 consultation cases. Among these, 68% of the consultations originated from our university and affiliated hospital. These cases covered diverse subject areas including medicine (49%), pharmacy (22%) and other fields. Furthermore, a total of 527 applications were submitted, out of which 398 were granted approval.

The TMUCRD has served as the foundation for a wide array of research endeavours. These studies have delved into various subjects, such as using machine learning techniques to predict outcomes for patients with cancer, investigating the clinical implications of diabetes, exploring advanced CKD and assessing adverse outcomes following major surgeries.^38–40^

### Collaborative research

Traditionally, many researchers and scholars work in isolation with their own data. However, we are actively transitioning towards a more collaborative and iterative approach to research. This shift improves result cross-validation and self-checking, bolstering research reliability. Additionally, we support pharmaceutical companies in postmarket surveillance, contributing to product evaluations and healthcare quality advancement.

Notably, TMU is Taiwan’s exclusive official member of the OHDSI initiative. OHDSI is committed to enhancing clinical medical data’s value through big data analysis and AI methods. It promotes multiparty research collaboration across various domains and addresses complex issues. A vital part of OHDSI’s mission is creating standardised CDMs to streamline data systems worldwide, ensuring consistency and comparability.

Starting from September 2020, the TMU CDC embarked on the OHDSI-CDM grafting project. With guidance from OHDSI headquarters, we organised a series of online conferences providing transnational technical support for the implementation of TMU-OHDSI OMOP CDM grafting. To date, we have actively participated in three large-scale multinational collaborative research projects. These projects encompass prognostic analysis of antihypertensive drug therapy, assessment of the effectiveness of anticoagulant drugs and the evaluation of cancer safety associated with H2 receptor antagonists.^41^

### Conclusion

Data are the cornerstone of scientific research, profoundly affecting research outcomes. The TMU CDC is unwavering in its dedication to enhancing data quality, valuing larger data volumes, longer data periods, authenticity, diversity, integrity, standardisation, accessibility, privacy and robust data governance.

We continually develop advanced data management systems, including data processing tools, cloud-based decision interfaces, data sampling options and cross-institutional data quality checks. Our focus is on creating specialised databases, integrating diverse healthcare data like inspection reports, patient records, nutrition assessments and more. These databases adhere to international OMOP-CDM standards, supporting research on topics such as COVID-19, dementia, stroke, lung cancer, diabetes and CKD, exemplifying our commitment to diverse and impactful healthcare research.

## Data Availability

All data relevant to the study are included in the article or uploaded as online supplemental information.
