# Supplementary material for: Taipei Medical University Clinical Research Database: a collaborative hospital EHR database aligned with international common data standards
Source: BMJ Health Care Inform. 2024 May 14;31(1):e100890. doi: 10.1136/bmjhci-2023-100890 (PMC11097871; doi:10.1136/bmjhci-2023-100890)
Supplement: Supplementary data [file bmjhci-2023-100890supp001.pdf]

Taipei Medical University Clinical Research Database (TMUCRD) – A Collaborative Hospital EHR Database Aligned with International Common Data Standards

Supplement

Table of Contents

Figure S1. TMUCRD Relationship Diagram 2
Table S1. Detailed information of laboratory tests from TMUCRD 3

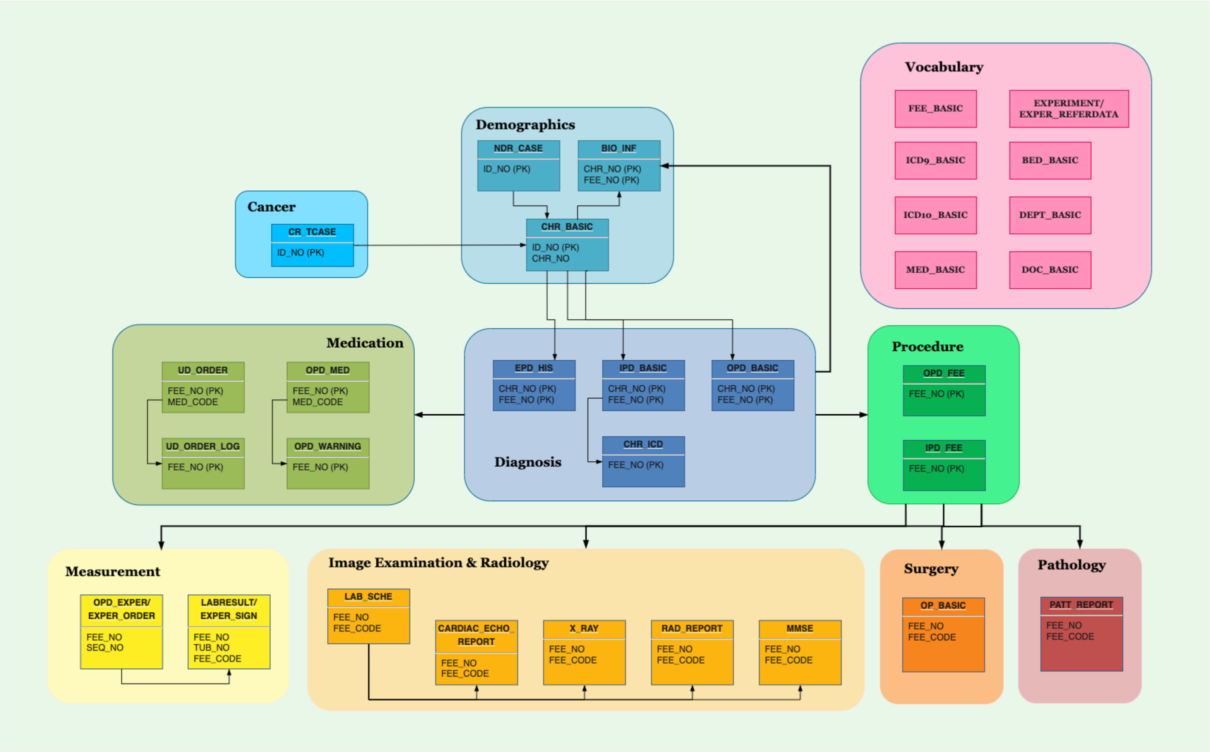

Figure S1. TMUCRD Relationship Diagram

Note: TMUCRD, Taipei Medical University Clinical Research Database;

Table S1. Detailed information of laboratory tests from TMUCRD

Year	WFH, n (million)	SHH, n (million)	TMUH, n (million)	#Patients, n (thousand)
**2004**	1.151	0	0	90.6
**2005**	1.010	0	0	84.8
**2006**	1.053	0	0	96
**2007**	1.138	0	0	98.2
**2008**	1.133	0.183	0	117.8
**2009**	1.200	0.724	0	159.9
**2010**	1.188	1.059	0	171.5
**2011**	1.011	1.280	0.062	162
**2012**	1.468	1.504	1.725	300.4
**2013**	1.557	1.670	1.963	315.5
**2014**	1.501	1.739	2.145	315.6
**2015**	2.825	1.873	2.335	350.9
**2016**	3.372	2.201	2.342	372.1
**2017**	3.428	2.485	2.384	377.6
**2018**	3.477	2.722	2.318	381.1
**2019**	3.636	3.025	2.277	392.1
**2020**	3.732	3.022	2.108	369.6
**2021**	3.799	3.038	2.208	359.2

Note: WFH, Wan-Fang Hospital; SHH, Shuang-Ho Hospital; TMUH, Taipei Medical University Hospital; TMUCRD, Taipei Medical University Clinical Research Database;
